# Supplementary material for: Effect of mulching and organic manure on maize yield, water, and nitrogen use efficiency in the Loess Plateau of China
Source: PeerJ. 2024 Dec 3;12:e18644. doi: 10.7717/peerj.18644 (PMC11623062; doi:10.7717/peerj.18644)
Supplement: Supplemental Information 2 [file peerj-12-18644-s002.doc]

**Supplementary Information (SI)**

**Exploration of organic farming: Effect of mulching and organic fertilizer rate on maize yield, water, and nitrogen use efficiency in the Loess Plateau of China**

Xing Yingying, Jintao Fu, Xiukang Wang^*^

*Key Laboratory of Applied Ecology of Loess Plateau, College of Life Science, Yan'an University, Yan'an, Shaanxi 716000, China*

* Corresponding author (Tel: +86-0911-2332030; Email: [wangxiukang@126.com](mailto:wangxiukang@126.com))

**Supplementary Table S1.** The correlation among the grain yield, biomass, biomass, harvest index, water use efficiency, nitrogen uptake in grain yield, nitrogen use efficiency, soil water content at former growth stage, soil water content at late growth stage, soil nitrate-N content at former growth stage, soil nitrate-N content at late growth stage using the average values in 2017 and 2018 (n=3).

| Item | GY | BM | HI | WUE | NUA | NUE | SWCF | SWCL | SNCF | SNCL |
| --- | --- | --- | --- | --- | --- | --- | --- | --- | --- | --- |
| SGY | 1 |  |  |  |  |  |  |  |  |  |
| BM | 0.948** | 1 |  |  |  |  |  |  |  |  |
| HI | 0.795** | 0.561 | 1 |  |  |  |  |  |  |  |
| WUE | 0.895** | 0.753** | 0.879** | 1 |  |  |  |  |  |  |
| NUA | 0.915** | 0.944** | 0.579* | 0.816** | 1 |  |  |  |  |  |
| NUE | -0.847** | -0.886** | -0.509 | -0.762** | -0.884** | 1 |  |  |  |  |
| SWCF | 0.229 | 0.129 | 0.366 | 0.1 | -0.113 | 0.023 | 1 |  |  |  |
| SWCL | 0.365 | 0.212 | 0.556 | 0.24 | 0.013 | 0.066 | 0.793** | 1 |  |  |
| SNCF | 0.777** | 0.793** | 0.526 | 0.512 | 0.656* | -0.494 | 0.455 | 0.609* | 1 |  |
| SNCL | 0.636* | 0.757** | 0.217 | 0.471 | 0.708** | -0.689* | 0.029 | 0.032 | 0.721** | 1 |

**Note:** GY, grain yield; BM, biomass; HI, harvest index; WUE, water use efficiency; NUA, nitrogen uptake in grain yield; NUE, nitrogen use efficiency; SWCF, soil water content at former growth stage; SWCL, soil water content at late growth stage; SNCF, soil nitrate-N content at former growth stage; SNCL, soil nitrate-N content at late growth stage; **. Correlation is significant at the 0.01 level (2-tailed); *. Correlation is significant at the 0.05 level (2-tailed).

**Supplementary Table S2**. Mean grain yield, biomass, biomass, harvest index, water use efficiency, nitrogen uptake in grain yield, nitrogen use efficiency, soil water content at former growth stage, soil water content at late growth stage, soil nitrate-N content at former growth stage, soil nitrate-N content at late growth stage in 2017.

| Item | GY | BM | HI | WUE | NUA | NUE | SWCF | SWCL | SNCF | SNCL |
| --- | --- | --- | --- | --- | --- | --- | --- | --- | --- | --- |
| T1 | 5760 | 18684 | 0.31 | 2.01 | 37.1 | 41.2 | 15.5 | 15.5 | 6.4 | 8.9 |
| T2 | 6320 | 20531 | 0.31 | 1.95 | 33.2 | 36.9 | 18.5 | 17.0 | 9.4 | 13.0 |
| T3 | 6752 | 22032 | 0.31 | 2.04 | 65.8 | 35.1 | 15.6 | 14.9 | 9.8 | 14.8 |
| T4 | 7465 | 22759 | 0.33 | 2.17 | 63.2 | 36.6 | 16.7 | 17.0 | 11.1 | 12.3 |
| T5 | 7667 | 23272 | 0.33 | 2.31 | 78.1 | 28.9 | 16.5 | 15.2 | 10.6 | 17.8 |
| T6 | 8296 | 23837 | 0.35 | 2.41 | 81.7 | 30.3 | 17.3 | 17.2 | 10.8 | 13.5 |

**Note:** GY, grain yield; BM, biomass; HI, harvest index; WUE, water use efficiency; NUA, nitrogen uptake in grain yield; NUE, nitrogen use efficiency; SWCF, soil water content at former growth stage; SWCL, soil water content at late growth stage; SNCF, soil nitrate-N content at former growth stage; SNCL, soil nitrate-N content at late growth stage.

**Supplementary Table S3**. Mean grain yield, biomass, biomass, harvest index, water use efficiency, nitrogen uptake in grain yield, nitrogen use efficiency, soil water content at former growth stage, soil water content at late growth stage, soil nitrate-N content at former growth stage, soil nitrate-N content at late growth stage in 2018.

| Item | GY | BM | HI | WUE | NUA | NUE | SWCF | SWCL | SNCF | SNCL |
| --- | --- | --- | --- | --- | --- | --- | --- | --- | --- | --- |
| T1 | 5952 | 18469 | 0.32 | 2.05 | 37.0 | 41.2 | 15.9 | 15.8 | 7.5 | 10.5 |
| T2 | 6437 | 19966 | 0.32 | 2.04 | 40.0 | 44.4 | 17.6 | 17.9 | 11.4 | 14.5 |
| T3 | 6881 | 22432 | 0.31 | 2.07 | 65.4 | 36.3 | 16.3 | 16.2 | 11.3 | 16.8 |
| T4 | 7482 | 22608 | 0.33 | 2.10 | 64.4 | 35.8 | 17.8 | 17.7 | 12.6 | 13.3 |
| T5 | 7681 | 23928 | 0.32 | 2.17 | 77.9 | 28.9 | 15.5 | 16.1 | 11.3 | 18.1 |
| T6 | 8422 | 24203 | 0.35 | 2.44 | 79.1 | 29.3 | 17.4 | 17.8 | 12.9 | 17.2 |

**Note:** GY, grain yield; BM, biomass; HI, harvest index; WUE, water use efficiency; NUA, nitrogen uptake in grain yield; NUE, nitrogen use efficiency; SWCF, soil water content at former growth stage; SWCL, soil water content at late growth stage; SNCF, soil nitrate-N content at former growth stage; SNCL, soil nitrate-N content at late growth stage.

**Supplementary Table S4**. The standardized values of grain yield, biomass, biomass, harvest index, water use efficiency, nitrogen uptake in grain yield, nitrogen use efficiency, soil water content at former growth stage, soil water content at late growth stage, soil nitrate-N content at former growth stage, soil nitrate-N content at late growth stage in 2017.

| Item | GY | BM | HI | WUE | NUA | NUE | SWCF | SWCL | SNCF | SNCL |
| --- | --- | --- | --- | --- | --- | --- | --- | --- | --- | --- |
| T1 | -1.369 | -1.643 | -0.817 | -0.764 | -1.115 | 1.400 | -1.031 | -0.618 | -1.907 | -1.532 |
| T2 | -0.772 | -0.685 | -0.765 | -1.105 | -1.305 | 0.456 | 1.620 | 0.850 | -0.148 | -0.125 |
| T3 | -0.311 | 0.093 | -0.882 | -0.580 | 0.292 | 0.063 | -0.973 | -1.191 | 0.084 | 0.471 |
| T4 | 0.450 | 0.470 | 0.387 | 0.136 | 0.165 | 0.379 | -0.014 | 0.828 | 0.809 | -0.360 |
| T5 | 0.665 | 0.736 | 0.464 | 0.872 | 0.892 | -1.298 | -0.178 | -0.879 | 0.526 | 1.512 |
| T6 | 1.337 | 1.029 | 1.614 | 1.442 | 1.071 | -1.001 | 0.576 | 1.009 | 0.637 | 0.034 |

**Note:** GY, grain yield; BM, biomass; HI, harvest index; WUE, water use efficiency; NUA, nitrogen uptake in grain yield; NUE, nitrogen use efficiency; SWCF, soil water content at former growth stage; SWCL, soil water content at late growth stage; SNCF, soil nitrate-N content at former growth stage; SNCL, soil nitrate-N content at late growth stage.

**Supplementary Table S5**. The standardized values of grain yield, biomass, biomass, harvest index, water use efficiency, nitrogen uptake in grain yield, nitrogen use efficiency, soil water content at former growth stage, soil water content at late growth stage, soil nitrate-N content at former growth stage, soil nitrate-N content at late growth stage in 2018.

| Item | GY | BM | HI | WUE | NUA | NUE | SWCF | SWCL | SNCF | SNCL |
| --- | --- | --- | --- | --- | --- | --- | --- | --- | --- | --- |
| T1 | -1.326 | -1.528 | -0.223 | -0.641 | -1.295 | 0.832 | -0.884 | -1.125 | -1.902 | -1.599 |
| T2 | -0.787 | -0.868 | -0.196 | -0.703 | -1.133 | 1.360 | 0.851 | 0.970 | 0.108 | -0.198 |
| T3 | -0.292 | 0.220 | -1.374 | -0.486 | 0.259 | 0.054 | -0.429 | -0.777 | 0.076 | 0.603 |
| T4 | 0.378 | 0.297 | 0.450 | -0.278 | 0.206 | -0.032 | 1.117 | 0.862 | 0.738 | -0.610 |
| T5 | 0.600 | 0.879 | -0.308 | 0.171 | 0.949 | -1.142 | -1.289 | -0.815 | 0.076 | 1.046 |
| T6 | 1.426 | 1.001 | 1.651 | 1.937 | 1.014 | -1.072 | 0.634 | 0.885 | 0.903 | 0.758 |

**Note:** GY, grain yield; BM, biomass; HI, harvest index; WUE, water use efficiency; NUA, nitrogen uptake in grain yield; NUE, nitrogen use efficiency; SWCF, soil water content at former growth stage; SWCL, soil water content at late growth stage; SNCF, soil nitrate-N content at former growth stage; SNCL, soil nitrate-N content at late growth stage.

**Supplementary Table S6.** The correlation matrix comes from the standardized values of grain yield, biomass, biomass, harvest index, water use efficiency, nitrogen uptake in grain yield, nitrogen use efficiency, soil water content at former growth stage, soil water content at late growth stage, soil nitrate-N content at former growth stage, soil nitrate-N content at late growth stage in 2017.

| Item | GY | BM | HI | WUE | NUA | NUE | SWCF | SWCL | SNCF | SNCL |
| --- | --- | --- | --- | --- | --- | --- | --- | --- | --- | --- |
| GY | 1 | 0.965 | 0.925 | 0.93 | 0.909 | -0.864 | 0.222 | 0.339 | 0.853 | 0.588 |
| BM | 0.965 | 1 | 0.794 | 0.83 | 0.918 | -0.883 | 0.19 | 0.22 | 0.933 | 0.725 |
| HI | 0.925 | 0.794 | 1 | 0.953 | 0.772 | -0.724 | 0.26 | 0.489 | 0.636 | 0.31 |
| WUE | 0.93 | 0.83 | 0.953 | 1 | 0.894 | -0.826 | 0.047 | 0.205 | 0.612 | 0.471 |
| NUA | 0.909 | 0.918 | 0.772 | 0.894 | 1 | -0.861 | -0.158 | -0.067 | 0.725 | 0.667 |
| NUE | -0.864 | -0.883 | -0.724 | -0.826 | -0.861 | 1 | -0.224 | -0.021 | -0.749 | -0.861 |
| SWCF | 0.222 | 0.19 | 0.26 | 0.047 | -0.158 | -0.224 | 1 | 0.78 | 0.381 | 0.135 |
| SWCL | 0.339 | 0.22 | 0.489 | 0.205 | -0.067 | -0.021 | 0.78 | 1 | 0.36 | -0.263 |
| SNCF | 0.853 | 0.933 | 0.636 | 0.612 | 0.725 | -0.749 | 0.381 | 0.36 | 1 | 0.701 |
| SNCL | 0.588 | 0.725 | 0.31 | 0.471 | 0.667 | -0.861 | 0.135 | -0.263 | 0.701 | 1 |

**Note:** GY, grain yield; BM, biomass; HI, harvest index; WUE, water use efficiency; NUA, nitrogen uptake in grain yield; NUE, nitrogen use efficiency; SWCF, soil water content at former growth stage; SWCL, soil water content at late growth stage; SNCF, soil nitrate-N content at former growth stage; SNCL, soil nitrate-N content at late growth stage.

**Supplementary Table S7.** The correlation matrix comes from the standardized values of grain yield, biomass, biomass, harvest index, water use efficiency, nitrogen uptake in grain yield, nitrogen use efficiency, soil water content at former growth stage, soil water content at late growth stage, soil nitrate-N content at former growth stage, soil nitrate-N content at late growth stage in 2018.

| Item | GY | BM | HI | WUE | NUA | NUE | SWCF | SWCL | SNCF | SNCL |
| --- | --- | --- | --- | --- | --- | --- | --- | --- | --- | --- |
| GY | 1 | 0.943 | 0.638 | 0.861 | 0.925 | -0.883 | 0.236 | 0.411 | 0.806 | 0.716 |
| BM | 0.943 | 1 | 0.345 | 0.698 | 0.986 | -0.905 | 0.07 | 0.226 | 0.804 | 0.849 |
| HI | 0.638 | 0.345 | 1 | 0.794 | 0.326 | -0.392 | 0.513 | 0.646 | 0.42 | 0.044 |
| WUE | 0.861 | 0.698 | 0.794 | 1 | 0.714 | -0.756 | 0.175 | 0.35 | 0.533 | 0.538 |
| NUA | 0.925 | 0.986 | 0.326 | 0.714 | 1 | -0.956 | -0.056 | 0.091 | 0.7 | 0.818 |
| NUE | -0.883 | -0.905 | -0.392 | -0.756 | -0.956 | 1 | 0.231 | 0.059 | -0.502 | -0.711 |
| SWCF | 0.236 | 0.07 | 0.513 | 0.175 | -0.056 | 0.231 | 1 | 0.945 | 0.608 | -0.113 |
| SWCL | 0.411 | 0.226 | 0.646 | 0.35 | 0.091 | 0.059 | 0.945 | 1 | 0.712 | 0.086 |
| SNCF | 0.806 | 0.804 | 0.42 | 0.533 | 0.7 | -0.502 | 0.608 | 0.712 | 1 | 0.676 |
| SNCL | 0.716 | 0.849 | 0.044 | 0.538 | 0.818 | -0.711 | -0.113 | 0.086 | 0.676 | 1 |

**Note:** GY, grain yield; BM, biomass; HI, harvest index; WUE, water use efficiency; NUA, nitrogen uptake in grain yield; NUE, nitrogen use efficiency; SWCF, soil water content at former growth stage; SWCL, soil water content at late growth stage; SNCF, soil nitrate-N content at former growth stage; SNCL, soil nitrate-N content at late growth stage.

**Supplementary Table S8**. Total variance explained of the contribution rate and accumulative of contribution rate with eigenvalues was calculated by principal component analysis in 2017.

| Component | Initial Eigenvalues | | | Extraction Sums of Squared Loadings | | | Rotation Sums of Squared Loadings | | |
| --- | --- | --- | --- | --- | --- | --- | --- | --- | --- |
|  | Total | % of Variance | Cumulative % | Total | % of Variance | Cumulative % | Total | % of Variance | Cumulative % |
| 1 | 6.608 | 66.077 | 66.077 | 6.608 | 66.077 | 66.077 |  |  | 0.66077 |
| 2 | 1.986 | 19.863 | 85.939 | 1.986 | 19.863 | 85.939 |  |  | 0.19863 |
| 3 | 1.025 | 10.249 | 96.188 | 1.025 | 10.249 | 96.188 |  |  | 0.10249 |
| 4 | 0.36 | 3.6 | 99.788 |  |  |  |  |  |  |
| 5 | 0.021 | 0.212 | 100 |  |  |  |  |  |  |
| 6 | 2.79E-16 | 2.79E-15 | 100 |  |  |  |  |  |  |
| 7 | 1.24E-16 | 1.24E-15 | 100 |  |  |  |  |  |  |
| 8 | 5.21E-17 | 5.21E-16 | 100 |  |  |  |  |  |  |
| 9 | -2.13E-16 | -2.13E-15 | 100 |  |  |  |  |  |  |
| 10 | -3.24E-16 | -3.24E-15 | 100 |  |  |  |  |  |  |

Extraction Method: Principal Component Analysis.

**Supplementary Table S9**. Total variance explained of the contribution rate and accumulative of contribution rate with eigenvalues was calculated by principal component analysis in 2017.

| Component | Initial Eigenvalues | | | Extraction Sums of Squared Loadings | | | Rotation Sums of Squared Loadings | | |
| --- | --- | --- | --- | --- | --- | --- | --- | --- | --- |
|  | Total | % of Variance | Cumulative % | Total | % of Variance | Cumulative % | Total | % of Variance | Cumulative % |
| 1 | 6.127 | 61.274 | 61.274 | 6.127 | 61.274 | 61.274 |  |  |  |
| 2 | 2.552 | 25.522 | 86.795 | 2.552 | 25.522 | 86.795 |  |  |  |
| 3 | 1.022 | 10.216 | 97.011 | 1.022 | 10.216 | 97.011 |  |  |  |
| 4 | 0.24 | 2.399 | 99.41 |  |  |  |  |  |  |
| 5 | 0.059 | 0.59 | 100 |  |  |  |  |  |  |
| 6 | 4.75E-16 | 4.75E-15 | 100 |  |  |  |  |  |  |
| 7 | 1.41E-16 | 1.41E-15 | 100 |  |  |  |  |  |  |
| 8 | 4.24E-17 | 4.24E-16 | 100 |  |  |  |  |  |  |
| 9 | -8.56E-18 | -8.56E-17 | 100 |  |  |  |  |  |  |
| 10 | -1.96E-16 | -1.96E-15 | 100 |  |  |  |  |  |  |

Extraction Method: Principal Component Analysis.

**Supplementary Table S10**. The three components extracted matrix by principal component analysis of grain yield, biomass, biomass, harvest index, water use efficiency, nitrogen uptake in grain yield, nitrogen use efficiency, soil water content at former growth stage, soil water content at late growth stage, soil nitrate-N content at former growth stage, soil nitrate-N content at late growth stage in 2017.

| Item | Component | | |
| --- | --- | --- | --- |
|  | 1 | 2 | 3 |
| GY | 0.987 | 0.064 | 0.139 |
| BM | 0.979 | -0.044 | -0.061 |
| HI | -0.929 | 0.173 | 0.196 |
| WUE | 0.915 | -0.35 | 0.169 |
| NUA | 0.906 | -0.062 | 0.369 |
| NUE | 0.875 | 0.145 | -0.287 |
| SWCF | 0.873 | 0.234 | 0.405 |
| SWCL | 0.709 | -0.373 | -0.587 |
| SNCF | 0.257 | 0.951 | 0.137 |
| SNCL | 0.244 | 0.839 | -0.435 |

**Note:** GY, grain yield; BM, biomass; HI, harvest index; WUE, water use efficiency; NUA, nitrogen uptake in grain yield; NUE, nitrogen use efficiency; SWCF, soil water content at former growth stage; SWCL, soil water content at late growth stage; SNCF, soil nitrate-N content at former growth stage; SNCL, soil nitrate-N content at late growth stage.

.

**Supplementary Table S11**. The three components extracted matrix by principal component analysis of grain yield, biomass, biomass, harvest index, water use efficiency, nitrogen uptake in grain yield, nitrogen use efficiency, soil water content at former growth stage, soil water content at late growth stage, soil nitrate-N content at former growth stage, soil nitrate-N content at late growth stage in 2018.

| Item | Component | | |
| --- | --- | --- | --- |
|  | 1 | 2 | 3 |
| GY | 0.994 | -0.015 | -0.079 |
| BM | 0.95 | -0.239 | 0.167 |
| HI | 0.921 | -0.353 | 0.062 |
| WUE | -0.855 | 0.457 | 0.193 |
| NUA | 0.851 | 0.045 | -0.45 |
| NUE | 0.846 | 0.318 | 0.426 |
| SWCF | 0.769 | -0.384 | 0.38 |
| SWCL | 0.601 | 0.522 | -0.597 |
| SNCF | 0.264 | 0.932 | 0.199 |
| SNCL | 0.441 | 0.877 | 0.147 |

**Note:** GY, grain yield; BM, biomass; HI, harvest index; WUE, water use efficiency; NUA, nitrogen uptake in grain yield; NUE, nitrogen use efficiency; SWCF, soil water content at former growth stage; SWCL, soil water content at late growth stage; SNCF, soil nitrate-N content at former growth stage; SNCL, soil nitrate-N content at late growth stage.

**Supplementary Table S12.** The score and rank of the comprehensive grain yield, harvest index, water use efficiency, and nitrogen use efficiency are calculated by use of principal component analysis method of all the treatments.

| Year | Treatment | Q1 | Q2 | Q3 | Q | Rank |
| --- | --- | --- | --- | --- | --- | --- |
| 2017 | T1 | -1.489 | -0.346 | 1.165 | -0.933 | 6 |
|  | T2 | -0.653 | 1.309 | -1.353 | -0.310 | 4 |
|  | T3 | -0.218 | -1.208 | -0.584 | -0.444 | 5 |
|  | T4 | 0.276 | 0.567 | 0.430 | 0.339 | 2 |
|  | T5 | 0.885 | -0.979 | -0.612 | 0.327 | 3 |
|  | T6 | 1.199 | 0.657 | 0.954 | 1.021 | 1 |
| 2018 | T1 | -1.456 | -0.283 | -1.378 | -1.160 | 6 |
|  | T2 | -0.643 | 1.121 | 0.809 | -0.119 | 4 |
|  | T3 | -0.172 | -0.840 | 1.163 | -0.161 | 5 |
|  | T4 | 0.283 | 0.910 | 0.308 | 0.400 | 2 |
|  | T5 | 0.558 | -1.380 | 0.096 | 0.104 | 3 |
|  | T6 | 1.431 | 0.473 | -0.998 | 0.937 | 1 |

**Supplementary Table S13.** The averages of the main effects of mulching and organic nitrogen on grain yield, biomass, and harvest index.

| Years | Nitrogen fertilizer (kg ha^-1^) | Mulching | Grain yield  (kg ha^-1^) | Biomass  (kg ha^-1^) | Harvest index |
| --- | --- | --- | --- | --- | --- |
| 2017 |  | Mulch | 7360.33 | 22375.67 | 0.33 |
|  |  | No mulch | 6726.33 | 21330.33 | 0.32 |
|  | 12000 |  | 6040.00 | 19609.00 | 0.31 |
|  | 24000 |  | 7108.50 | 22395.50 | 0.32 |
|  | 36000 |  | 7981.50 | 23554.50 | 0.34 |
| 2018 |  | Mulch | 7447.00 | 22259.00 | 0.33 |
|  |  | No mulch | 6838.00 | 21609.67 | 0.32 |
|  | 12000 |  | 6194.50 | 19217.50 | 0.32 |
|  | 24000 |  | 7181.50 | 22520.00 | 0.32 |
|  | 36000 |  | 8051.50 | 24065.50 | 0.33 |

**Supplementary Table S14.** The averages of the main effects of mulching and organic nitrogen on grain yield, biomass, and harvest index.

| Years | Nitrogen fertilizer (kg ha^-1^) | Mulching | WUE (kg m^-3^) | Nitrogen uptake (kg ha^-1^) | NUE (%) |
| --- | --- | --- | --- | --- | --- |
| 2017 |  | No | 2.12 | 58.16 | 33.64 |
|  |  | Yes | 2.18 | 61.53 | 36.01 |
|  | 12000 |  | 1.98 | 35.15 | 39.06 |
|  | 24000 |  | 2.11 | 64.51 | 35.84 |
|  | 36000 |  | 2.36 | 79.88 | 29.59 |
| 2018 |  | No | 2.10 | 60.12 | 35.45 |
|  |  | Yes | 2.19 | 61.18 | 36.51 |
|  | 12000 |  | 2.05 | 38.53 | 42.81 |
|  | 24000 |  | 2.09 | 64.89 | 36.05 |
|  | 36000 |  | 2.31 | 78.54 | 29.09 |
